# Time-Response-Histogram-Based Feature of Magnetic Barkhausen Noise for Material Characterization Considering Influences of Grain and Grain Boundary under In Situ Tensile Test

**DOI:** 10.3390/s21072350

**Published:** 2021-03-28

**Authors:** Jia Liu, Guiyun Tian, Bin Gao, Kun Zeng, Yongbing Xu, Qianhang Liu

**Affiliations:** 1 School of Automation Engineering, University of Electronic Science and Technology of China, Chengdu 611731, China; bin_gao@uestc.edu.cn (B.G.); kunzeng@uestc.edu.cn (K.Z.); 201921060635@std.uestc.edu.cn (Q.L.); 2 School of Engineering, Newcastle University, Newcastle upon Tyne NE1 7RU, UK; 3 Spintronics and Nanodevice Laboratory, Department of Electronics Engineering, University of York, York YO10 5DD, UK; yongbing.xu@york.ac.uk

**Keywords:** time-response histogram, magnetic Barkhausen noise, stress evaluation, grain/grain boundary, domain-wall motion

## Abstract

Stress is the crucial factor of ferromagnetic material failure origin. However, the nondestructive test methods to analyze the ferromagnetic material properties’ inhomogeneity on the microscopic scale with stress have not been obtained so far. In this study, magnetic Barkhausen noise (MBN) signals on different silicon steel sheet locations under in situ tensile tests were detected by a high-spatial-resolution magnetic probe. The domain-wall (DW) motion, grain, and grain boundary were detected using a magneto-optical Kerr (MOKE) image. The time characteristic of DW motion and MBN signals on different locations was varied during elastic deformation. Therefore, a time-response histogram is proposed in this work to show different DW motions inside the grain and around the grain boundary under low tensile stress. In order to separate the variation of magnetic properties affected by the grain and grain boundary under low tensile stress corresponding to MBN excitation, time-division was carried out to extract the root-mean-square (RMS), mean, and peak in the optimized time interval. The time-response histogram of MBN evaluated the silicon steel sheet’s inhomogeneous material properties, and provided a theoretical and experimental reference for ferromagnetic material properties under stress.

## 1. Introduction

Stresses play a pivotal role in determining the durability and service life of components. [1,2,3]. Jun et al. reported that components are vulnerable because of crack initiation caused by the generation of high tensile stresses [4]. Lee et al. assessed the fatigue life of a welded repaired rail based on the welding process and contact stresses [5].

In most cases, the material state evaluation is considered to be homogeneous in the macrolevel analysis; however, stress-measurement research is necessary to connect with the microstructural properties on the grain scale. Donegan et al. used a convolutional neural network to predict which regions of a microstructure are susceptible to forming stress concentration and serving areas of local damage accumulation [6]. Zarei et al. found that by considering the various orientations and shapes of the grains, the finite element model of DP steel microstructure results in a better prediction of the stress–strain curve than the homogeneous models [7]. Zhao et al. proposed an automatic 3D simulation to simulate metallic materials’ microstructure and investigated each grain’s stress for polycrystalline material [8]. Johnson et al. used high-resolution electron backscatter diffraction to show the intersection between material properties’ variation and grain boundaries for analysis of the crack-initiation process [9]. Analysis of the stress state affected by the grain and grain boundary is usually carried out in a simulation or a destructive manner [10]. For this reason, it would be beneficial to propose a reliable nondestructive technique designed for the purpose above.

The variation of the nondestructive test and evaluation method is used to characterize the electromagnetic properties for industrial materials’ state evaluation. Among them, magnetic Barkhausen noise (MBN) is a microstructure-sensitive and nondestructive evaluation technique for online inspection of structures to characterize both micro- and macrostresses of ferromagnetic material in industrial applications [11,12]. The MBN signal is found in the domain-wall (DW) motion, detected by a pick-up coil wound around the ferromagnetic sample [13,14]. The microstructure and the stress affect the alignment of the DWs, whereas lattice defects drive the free path of DW motion [15,16,17]. Nondestructive MBN determines both micro-and macroresidual stresses in silicon steel, steel weldments, and lightly deformed AISI 1070 steel [18,19,20,21].

MBN signals are combined with microstructure observation methods performed using magneto-optical Kerr (MOKE), atomic force microscopy (AFM), scanning electron microscopy (SEM), etc., to analyze the relationship between the ferromagnetic material’s microstructure and mechanical properties [22,23]. The peak value of MBN signals is calibrated as a function of the microstructure expressed in dislocation density [24]. MBN distinguishes the variable character of the microstructure and stress state for tool steel X210Cr12 [25]. The arrangement of magnetic domains along a grain boundary can be affected by the grain-boundary characteristic, and the MBN signal increased when increasing the angle of the grain-boundary misorientation [26]. The stress dependence on the MBN voltage and on the magnetic hysteresis loop is influenced by the grain size of commercial carbon steel [27]. Due to the MBN phenomenon observed in various directions for grain-oriented steels, MBN time-frequency distribution can analyze anisotropy distribution of the sample [28].

However, the effect of microstructure on material properties’ inhomogeneity is the key for the microscopic nondestructive stress characterization of the ferromagnetic material [29]. In particular, there is no reliable method for evaluating micromagnetic properties affected by the grain interior and grain boundary with different average stress via MBN.

As mentioned above, the microstructure and MBN of silicon steel sheet under an in situ tensile test were detected by MOKE and MBN detection device in this study. By combining the time characteristic of DW motion and MBN signal on different locations, a time-response histogram of MBN was carried out to quantitatively analyze the effect of the grain and grain boundary on the magnetic properties’ inhomogeneity during elastic deformation. The optical time intervals were used to separate the variation of magnetic properties affected by the grain interior and grain boundary. The proposed work will analyze the inhomogeneous material properties affected by the stress, grain, and grain boundary for the ferromagnetic material.

## 2. Experimental Procedure and Methods

MBN signals of a silicon steel sheet were detected by high-spatial-resolution sensing. Combining DW motion and MBN signals, a time-response histogram was carried out to evaluate the inhomogeneous magnetic properties with different stress inside the grain and around the grain boundary.

### 2.1. Sample Preparation

The grain-oriented silicon steel sheet was investigated to analyze the correlation of DW motion and the time-response histogram inside the grain and around the grain boundary with stress state. The average grain size of the selected silicon steel sheet was over 10 mm, which is visible for DW motion and MBN signal detection on grains and grain boundaries. The dimension of the sample was 300 mm × 30 mm × 0.2 mm (length × width × thickness). The sample was subjected to a tensile load. Grain-oriented silicon steel has the characteristics of high magnetic induction and low iron loss [30,31]. The chemical composition of the silicon steel sheet is found in Table 1. In order to observe microstructure and DW motion by MOKE, the sample was mechanically polished, and annealing treatment was not performed before the tensile test. Therefore, the initial state of the sample surface’s thin layer contained small compressive stresses.

### 2.2. Experiment Setup

**Figure 1:**
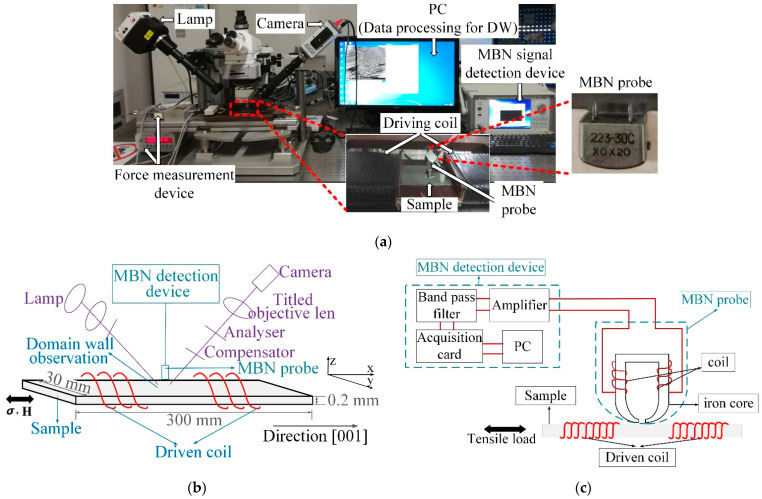
(**a**) Experimental set-up for DW and MBN observation; (**b**) schematic illustration of load, DW observation, and MBN detection; and (**c**) schematic for high-spatial-resolution MBN probe (about 3–4 mm) and MBN detection device.

The experimental setup for the MOKE and MBN observation for inhomogeneous material properties under the in situ tensile test is shown in Figure 1. MBN and longitudinal MOKE microscopy captured the MBN signal and DW motion of the silicon steel sheet. This system included a high-spatial-resolution MBN probe, an MBN detection device (including signal amplifiers, filter, acquisition card, MBN signal processor), a digital CCD camera for DW observation, driving coils, and applied tensile stress [32]. The tensile stress was from 0 MPa to 123 MPa, which was in the elastic deformation. A C8484-03G02 digital CCD camera with a sampling rate of 16.3 Hz was used to observe the microstructure and DW motion directly. DW motion images and MBN signals were in the same excitation, which could bridge the correlation of the micro- and macromagnetic parameters and stress inside the grain and around the grain boundary. The applied excitation field was in the tensile stress direction. The driven coil with 355 turns wound around the sample could provide AC to magnetize the sample for periodicity. The excitation was a triangular wave, in which the magnetization frequency and amplitude were 0.5 Hz and 1 kA/m, respectively. This magnetic field strength was greater than the sample material’s maximum magnetic saturation level (about 0.9 kA/m). The frequency spectrum of MBN signals generated over different ranges of magnetization did not change significantly [33,34]. Since the MBN signal generated in the material was attenuated by the electromagnetic eddy current opposition to an extent that depended on the frequency of the signal, the measurement depth (skin-depth) was limited to a finite depth from the surface [33]. The electromagnetic skin-depth δ is given by the relation:

(1) δ=1(πfσμ0μr)

where f is the frequency, σ is the conductivity of the material, $\mu_{0}$ is the permeability of vacuum, and $\mu_{r}$ is the relative permeability of the material. The frequency response strongly influences the maximum depth over which the changes in magnetization are detected as MBN signals. The magnetic probe with a dominant low-frequency response has greater skin-depth of detection, while the magnetic probe with more high-frequency response has shallow skin-depth. In this study, the thickness of the silicon steel sheet was 0.2 mm. Therefore, the magnetic probe could detect the MBN signal for the entire thickness of the sheet, when the frequency of the sample was 0.5 Hz. The time-response histogram was based on the correlation between the applied field and the DW motion at different locations.

The magnetic domain image was about 16.2 mm × 13.6 mm with 450 × 360 pixels, showing the microstructure and DW motion directly. The tape-recorder head was used for MBN detection. The probe was actually a hoof-shaped electromagnet. The probe was made by winding a coil around an iron core. When the excitation was applied to the sample, the probe read the data by sensing the magnetization on the sample. The probe had a high spatial resolution (about 3–4 mm), which could detect the different magnetization of the sample inside the grain and around the grain boundary. Linking the micromagnetic properties observed by MOKE, this system analyzed the difference of the time-response histogram of MBN affected by the interior and grain boundary, which interpreted the effect of the grain interior and grain boundary on the inhomogeneous material’s properties with different average tensile stress.

### 2.3. The Model of the Time-Response Histogram

**Figure 2:**
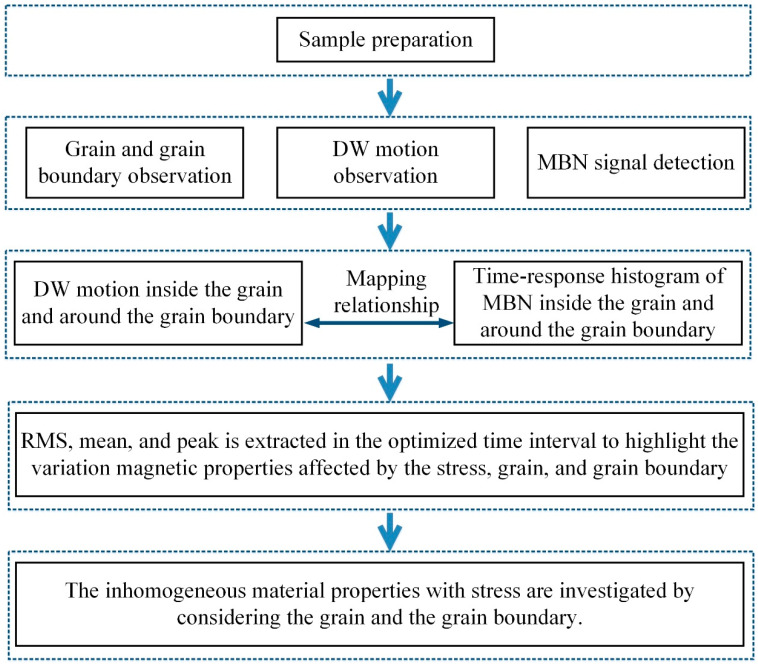
The approach of the time-response histogram to evaluate the inhomogeneous material’s properties on the grain and grain boundary.

The method for the time-response histogram on the grain and grain boundary with different stress state to analyze the effect of grain interior and grain boundary on the inhomogeneous material’s properties is outlined in Figure 2.

The microstructure and MBN signal of the silicon steel sheet were observed using a MOKE and MBN detection device. A tape-type probe was also designed as an MBN sensor to get the high spatial resolution of the MBN signal inside the grain and around the grain boundary. The combination of DW motion and MBN can analyze inhomogeneous material properties while considering the influence of the grain and grain boundary under tensile stress.

**Figure 3:**
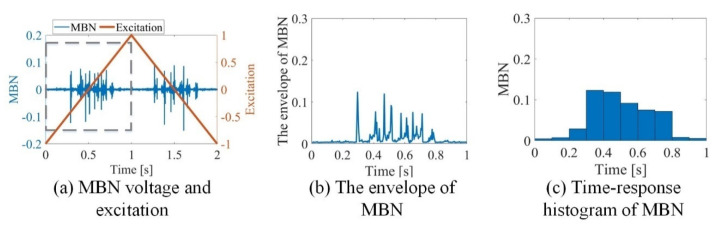
(**a**) Exemplary distributions of MBN voltage and excitation inside the grain acquired for excitation frequency of 10 Hz and amplitude of 1 kA/m; (**b**) the envelope of MBN extracted from the rectangular region of Figure 3a; (**c**) time-response histogram of MBN extracted from the rectangular region of Figure 3a.

Grain boundaries are important microstructural elements that affect DW motion, leading to a change in the differential susceptibility at all field strengths, thereby changing the time characteristics of MBN [35,36]. The time-response histogram was proposed to divide the entire time range of MBN activities into a series of equal intervals, showing the time characteristics of DW motion and MBN at different locations with stress along time. As shown in Figure 3, the time-response histogram was extracted from the MBN signal. Thus, the time-response histogram was modeled to analyze the variation of magnetic properties affected by the grain and grain boundary with different stress.

The mean, RMS, and peak were extracted from the time-response histogram to quantify the time characteristics of magnetic properties at different positions. Based on the correlation among the applied field and magnetic time characteristics on the grain and grain boundary, time-division was carried out to analyze the time-response histogram features in the optimized time intervals. The difference between the RMS, mean, and peak with tensile stress and without tensile stress was determined to reduce the difference of the time-response histogram before the tensile test. The feature extraction and time-division quantitatively analyzed the inhomogeneous material’s properties affected by the grain, grain boundary, and stress.

Therefore, these methods analyzed the silicon steel’s inhomogeneous material properties affected by the stress, grain, and grain boundary.

## 3. Experimental Results

To analyze the influence of grain interior and grain boundary on the inhomogeneous magnetic properties, the results of the time-response histogram were investigated to analyze the variation of MBN at different locations.

### 3.1. The Relationship between the Time-Response Histogram and DW Motion on the Grain and Grain Boundary

**Figure 4:**
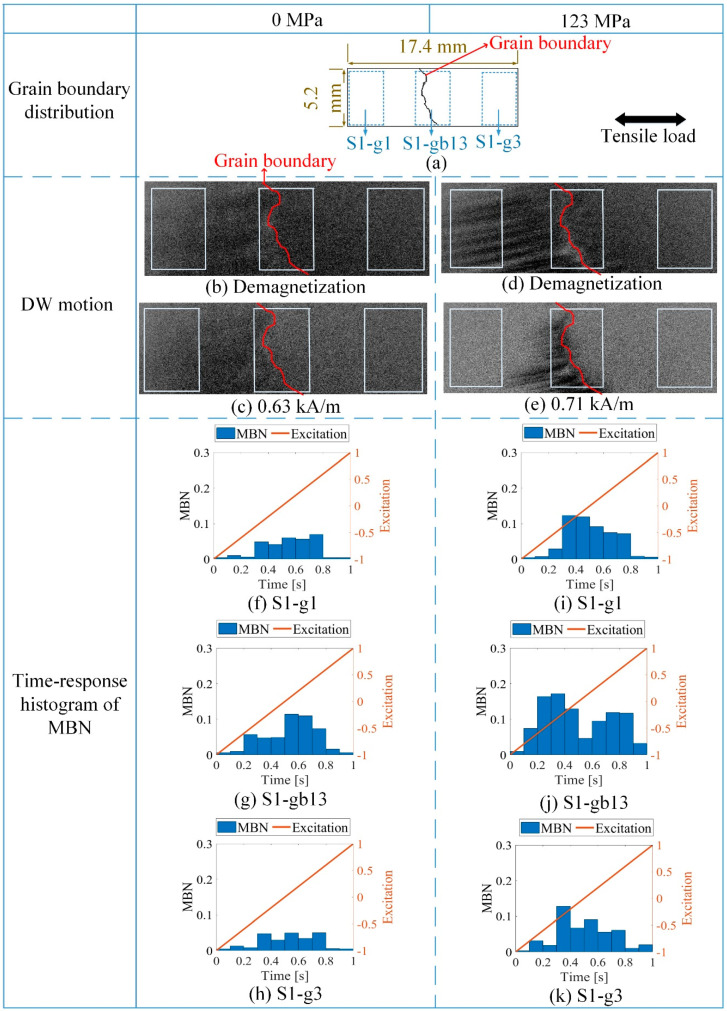
Different time-response histograms of the sample at different locations when the tensile stresses were 0 MPa and 123 MPa, respectively: (**a**) denotes the locations of S1-gb13, S1-g1, and S1-g3; (**b**,**c**) show the DW motion at different locations under 0 MPa, (**d**,**e**) show different DW motion on the different locations under 123 MPa; (**f**–**h**) show the related similar time-response histograms of MBN under 0 MPa; (**i**–**k**) show the quite different time-response histograms of MBN under 123 MPa.

Based on the observation of grain distribution and MBN of silicon steel, the time-response histogram was used to investigate the influence of the grain and grain boundary on the micromagnetic properties under tensile stress. In Figure 4, when the tensile stress was 0 MPa or 123 MPa, respectively, DW motion, hysteresis loops, and the time-response histogram were analyzed inside the grain and around the grain boundary. The distribution of the grain and grain boundary is shown in Figure 4a; S1-g1 and S1-g3 denote two adjacent grains, and S1-gb13 denotes the grain boundary between grains S1-g1 and S1-g3.

The magnetic domain distribution is predicted through the minimization of general energy relations [37]:

(2) $$E_{total}=E_{mag}+E_{ex}+E_{a}+E_{\sigma }$$

where $E_{mag}$, $E_{ex}$, $E_{a}$, and $E_{\sigma }$ respectively denote the magnetostatic energy, exchange energy, magnetocrystalline energy, and magnetoelastic and magnetostatic energies. The specific energy components are defined by:

(3) $$E_{mag}=-\frac{1}{2}\sum_{x}\sum_{y}\sum_{z}m_{i}\cdot H_{I}$$

(4) $$E_{ex}=-2\sum_{x}\sum_{y}\sum_{z}JS_{i}\cdot S_{j}$$

(5) $$E_{a}=-2\sum_{x}\sum_{y}\sum_{z}\left[K_{1}\left(\alpha_{1}{}^{2}\alpha_{2}{}^{2}+\alpha_{2}{}^{2}\alpha_{3}{}^{2}+\alpha_{3}{}^{2}\alpha_{1}{}^{2}\right)+K_{2}\alpha_{1}{}^{2}\alpha_{2}{}^{2}\alpha_{3}{}^{2}\right]$$

(6) $$E_{\lambda }=-\frac{3}{2}\sum_{x}\sum_{y}\sum_{z}\lambda \sigma \cos^{2}\theta$$

where $K_{1}$ and $K_{2}$ are anisotropy constants, and $\alpha_{1}$, $\alpha_{2}$, and $\alpha_{3}$ are direction cosines of a moment at the center of the cube. Furthermore, λ, σ, and θ respectively denote the magnetostriction constant, magnetoelastic stress and angle between the magnetization and stress. Finally, $H_{I}$ denotes the interaction field at $m_{i}$ due to all magnetic moment.

Magnetic total energies change with the increasing low tensile stresses inside grains and around grains boundaries. These changes affect the distribution of the magnetic domain. The equilibrium size of the magnetic domains is determined by minimizing the magnetic total energies. One possible way to avoid or reduce this energy is to increase the volume of the main domains with magnetization parallel to one of the easy axes and reduce the volume of domains magnetized along the other axes [37]. When low tensile stress is applied to the material, the increased magnetoelastic energy can be reduced by the formation of adapted domain patterns in the grain interior and around the grain boundary.

The grain interior has the same magnetic domain-wall distribution, on the assumption that the tensile stress and material magnetic properties are uniform with a finite size. Grain boundaries can affect the domain-wall distribution in polycrystalline materials, since grain boundaries are composed entirely of lattice defects [38]. Due to the differences in microstructure among grains and grain boundaries, when low tensile stresses are applied to the material, the changes of the magnetoelastic energy are different inside grains and around grains boundaries.

The microstructure and stress can affect the distribution of the magnetic domain and the behavior of DW motion around the grain boundary [39]. When the tensile stress is 0 MPa, DW motion is almost synchronous. Tensile stress aligns the magnetic domains parallel to the stress direction and makes the magnetization process easy when excitation is in the stress direction during the elastic deformation [40]. The formation of 180° DW increases inside the grain. The stable state of the magnetic domain structure is reached because the magnetic domain’s orientation makes the fewer free pole appear on the grain interface to reduce the magneto-static energy [41,42]. The 90° DWs appeared on the grain boundary S1-gb13 under 123 MPa to minimize the magnetic–static energy on the grain boundary affected by the stress. Thus, the distribution of DW was more complex on grain boundary S1-gb13 than on grains S1-g1 and S1-g3. Since stress made DW distribution more complex on the grain boundary, the DW motion became different at different locations with stress.

The DW motion in magnetization occurring over a given time interval relates to MBN activity [43,44]. In particular, the change rate of magnetization occurring as Barkhausen emissions $dM_{JS}/dt$ is expected to be proportional to the change rate of magnetization occurring as magnetization changes dM/dt. The time-response histogram was modeled to divide the entire time range of MBN activities into a series of equal intervals. Each bin of the histogram is represented as the magnetization change with the dimensionless term. The time characteristic of Barkhausen noise is distributed as:

(7) $$\frac{dM_{JS}}{dt}=\gamma \frac{dM}{dH}\frac{dH}{dt}$$

where H denotes the external magnetic field. In this study, the time-response histogram identified the amplitude of MBN activity along time.

The grain boundary affects the movement of DWs and leads to different magnetization processes under stress in the previous research [45]. From Figure 4d,e, there were more $90^{{}^{\circ}}$ domain walls around the grain boundary S1-gb13 during magnetization, and the time required to make material achieve saturation was longer around the grain boundary than inside the grain. The relationship between the difference of MBN activity $\Delta M_{JS}$, and the difference of magnetization intensity ΔM on different locations satisfies:

(8) $$\frac{d\Delta M_{JS}}{dt}\propto \gamma \frac{d\Delta M}{dH}\frac{dH}{dt}$$

When the tensile stress was 0 MPa, the difference in the time-response histograms for S1-g1, S1-g3, and S1-gb13 was small. When the tensile stress increased, MBN signals at these locations became much larger. The time-response histogram bins were quite different. When the tensile stress was 123 MPa, the histogram on S1-g3 was the smallest, and the histogram bins were the largest around the grain boundary S1-gb13 compared to grains S1-g1 and S1-g3. When the tensile stress was 123 MPa, the magnetic saturation was reached earliest on grain S1-g2 (from 0.3 s to 0.7 s), and magnetic saturation was reached latest on the grain boundary S1-gb12 under 123 MPa (from 0.1 s to 0.9 s). The different DW motion affected the time-response histogram at different locations. The magnetization of polycrystalline ferromagnetic materials is a complex phenomenon. However, there is a consensus about some of its main stages in magnetic materials: domain rotation, domain nucleation, $180^{{}^{\circ}}$ DW motion, and $90^{{}^{\circ}}$ DW motion [46]. From Figure 4, it was found that the pure $180^{{}^{\circ}}$ domain-wall motion was inside the grain interior (from 0.3 s to 0.7 s), and the $90^{{}^{\circ}}$ domain walls were associated with the bins from 0.1 s to 0.3 s and from 0.7 s to 0.9 s around the grain boundary.

Thus, the time-response histogram of MBN characterized the different DW motion inside the grain and around the grain boundary. Each bin of the time-response histogram $V_{n}$ was proportional to the DW motion velocity $v_{DW}$ [35], where n denotes the number of histogram bins. The relationship between the DW motion and time-response histogram’s bin at different locations satisfies:

(9) $$\Delta V_{n}\left(\sigma \right)\propto \Delta v\left(\sigma \right)$$

where $\Delta V_{n}\left(\sigma \right)$ and Δv(σ) denote the difference of the time-response histogram’s bin and the difference of DW motion velocity under stress on the grain and grain boundary. Based on studying the DW motion at different locations, the time-response histogram quantifies the micromagnetic, reflecting the variation of magnetic properties on the grain and grain boundary under stress.

### 3.2. Verification for Time-Response Histogram on Different Grains and Grain Boundaries

To further analyze the influence of grain and grain boundary on material properties, the variation of the time-response histogram at different locations under the tensile test was analyzed.

**Figure 5:**
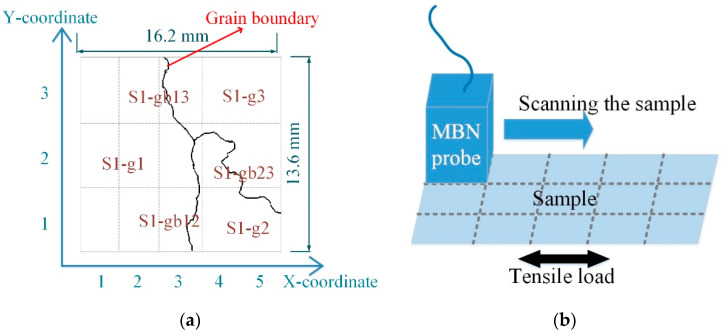
The sample was divided into 15 parts (5×3) by a dotted grid: (**a**) illustrates the grain distribution of the sample and the coordinate of each part; (**b**) shows MBN detection in each part by scanning the sample. S1-g1, S1-g2, and S1-g3 denote three adjacent grains of the sample; S1-gb12 denotes the grain boundary between grain S1-g1 and grain S1-g2; S1-gb13 denotes the grain boundary between grain S1-g1 and grain S1-g3; and S1-gb23 denotes the grain boundary between grain S1-g2 and grain S1-g3.

Figure 5 shows the grain and the grain boundary distribution of the sample. The sample was divided into 15 parts (5×3) by a dotted grid. The area of each grid area was 3.24 mm×4.53 mm. The X-coordinate and Y-coordinate defined each part’s location, as shown in Figure 5a. The size of the MBN probe was about 3–4 mm. The MBN signals were detected around the grain boundary when the MBN probe was on the grain boundaries S1-gb13 and S1-gb23. Due to the high-spatial-resolution magnetic probe, the time-response histogram of MBN was detected on each part by scanning the sample, as shown in Figure 5b. The magnetic domain and MBN were analyzed to investigate material properties at different locations.

**Figure 6:**
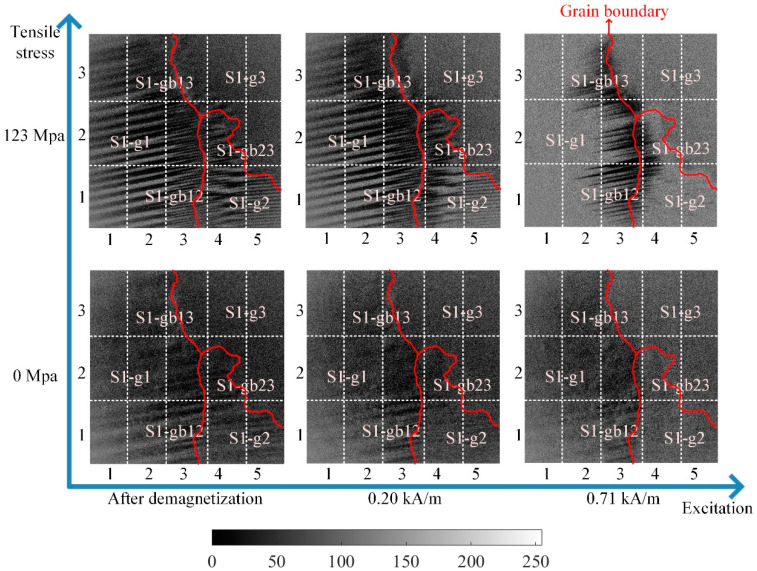
The magnetic domain and DW motion inside the grain and around the grain boundary of the sample with the different tensile stresses. The stress was increased from 0 MPa to 123 MPa. When the tensile stress increased, the difference of DW motion increased on the grain and grain boundary.

**Figure 7:**
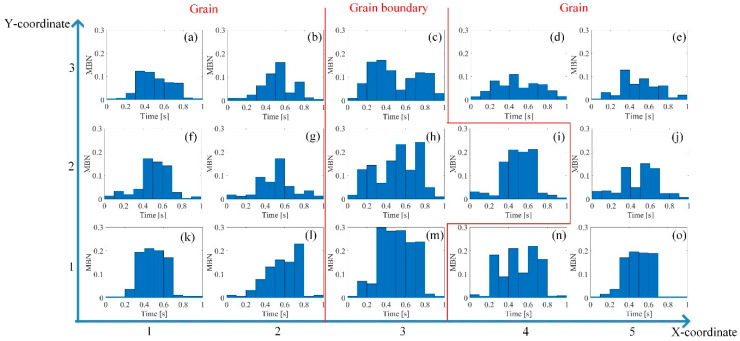
Time-response histograms at different locations when the tensile stress was 123 MPa. The time-response histograms increased with the increase of tensile stress, and the difference in MBN histograms on each part became much larger.

**Figure 8:**
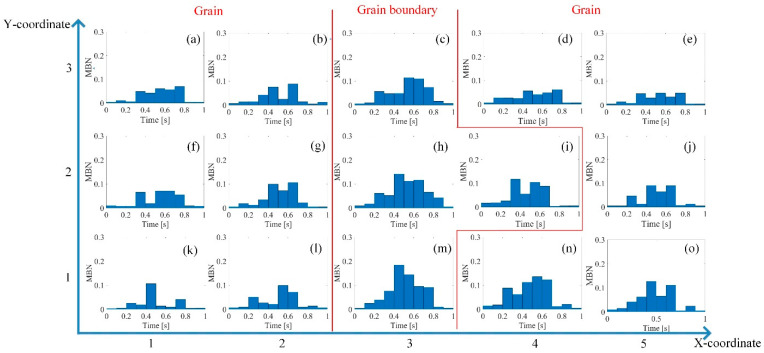
Time-response histograms at different locations when the tensile stress was 0 MPa. The time-response histograms exhibited a small difference before the tensile test. The difference was influenced by the material manufacturing.

DW motion varied with the increased stress at different locations. Figure 6 illustrates the different DW motions inside the grain and around the grain boundary under 0 MPa and 123 MPa. The difference of the DW motion on the grain and the grain boundary under 0 MPa was small, while the DW motions become quite different at different locations with the increase of stress. When the average tensile stress was 123 MPa, the DW distribution was more complicated on the grain boundary than on the grain. From Figure 6, the $90^{{}^{\circ}}$ DWs formed around grain boundary S1-gb13 and S1-gb12 to reduce the increasing total energy caused by the tensile stress. Especially on S1-gb12, magnetic field lines were mostly continuous across the grain boundary to reduce free poles on the grain interface under 123 MPa. Due to increasingly complex domain reorganization around S1-gb12, the energy loss was higher at this location, so these phenomena satisfy Equation (2) [47]. Thus, the MBN activities inside the grain and around the grain boundary became different with the increase of stress. The time-response histograms of MBN were different. Figure 7 and Figure 8 show the time-response histograms on every part under the tensile test. Figure 7a,b,d–g,j–m,n,o and Figure 8a,b,d–g,j–l,n,o denoted the time-response histogram inside the grain under 123 MPa and 0 MPa, while Figure 7c,h,i,m and Figure 8c,h,i,m denoted the time-response on the grain boundary under 123 MPa and 0 MPa. From Figure 8, when tensile stress was 0 MPa, since the difference of DW motion was tiny inside the grain and around the grain boundary, the difference between the time-response histograms at these locations was tiny (see Figure 8a,b,d–g,i–l,n,o). The tiny difference in the time-response histograms was caused by the sample surface’s mechanical polishing and material manufacturing. When the tensile stress increased, the time-response histograms became much larger, and the difference in the histograms at different locations became much larger. Since the strength of the external magnetic field required to make the material achieve saturation magnetization was much larger on the grain boundary (S1-gb12 and S1-gb13) than on the grains (S1-g1, S1-g2, and S1-g3), the amplitude of the time-response histograms became bigger on the grain boundary (S1-gb12 and S1-gb13) than on the grain (S1-g1, S1-g2, and S1-g3). The results shown in Figure 6, Figure 7 and Figure 8 are consistent with the results shown in Figure 4.

The high-spatial-resolution MBN probe was sensitive enough for magnetic-property characterization, reflecting the grain and the grain boundary’s material properties. The time-response histogram for each part was consistent with the DW motion results, proving the reliable repeat of correlation between the DW motion and time-response histogram inside the grain and around the grain boundary. The material properties were inhomogeneous at different locations, so the grain boundary and adjacent grains affected mechanical behavior [8,48]. The time-response histogram of MBN had enough sensitivity to characterize the grain and grain boundary effect on the inhomogeneous material’s properties.

## 4. Discussion

In order to quantitatively analyze the different inhomogeneous magnetic properties at different locations with stress, RMS, mean, and peak value were extracted from the time-response histograms. Based on the time characteristics of MBN inside the grain and around the grain boundary under stress, time-division was carried out to extract RMS, mean, and peak in the optimized time interval, which highlighted the variation of magnetic properties affected by the stress, grain, and grain boundary.

### 4.1. Time-Response Histogram’s Feature Extraction with Different Stress

The time-response histogram of MBN reflected the variation of the material state at different locations. MBN characteristic parameters such as RMS, mean value, and peak value had a high correlation with stress in previous studies [28]. RMS, mean, and peak value on the grains and grain boundaries were extracted from the histograms to investigate their statistical properties, which quantitatively analyzed the material properties. In this study, the magnetization frequency was 0.5 Hz. The interval of the time-response histogram bin was 0.1 s. The number of histogram bins was 10. Thus, the feature exaction for the quantitative method can be defined as:

(10) $$RMS\left(\sigma \right)=\sqrt{\frac{\sum_{i=1}^{N}V_{n}(\sigma )}{N}}$$

(11) $$Peakvalue\left(\sigma \right)=max\left(V_{n}\left(\sigma \right)\right)$$

(12) $$mean\left(\sigma \right)=\bar{V_{n}\left(\sigma \right)}$$

where N denotes the number of histogram bins and $V_{n}\left(\sigma \right)$ denotes the amplitude of each bin.

**Figure 9:**
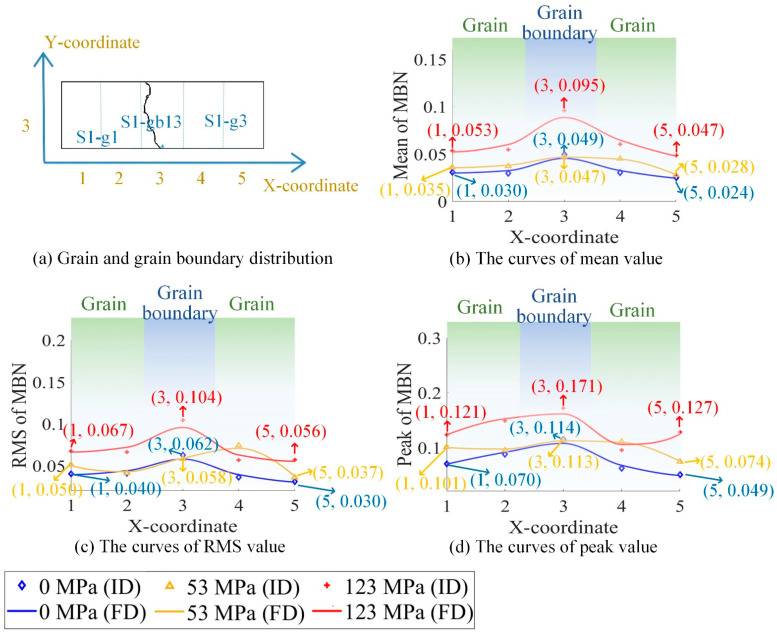
The curves for the RMS, mean, and peak value of time-response histograms under stress, reflecting the inhomogeneity of magnetic properties at different locations. FD denotes the fitting data, and ID denotes the original data.

**Figure 10:**
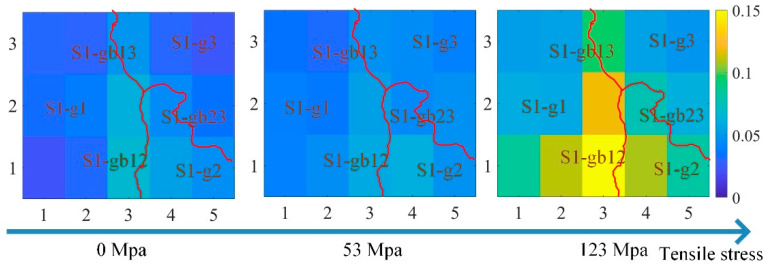
The images for the mean of the time-response histograms at different locations to reflect the inhomogeneous magnetic properties at different locations. The size of each image is 16.2 mm × 13.6 mm.

**Figure 11:**
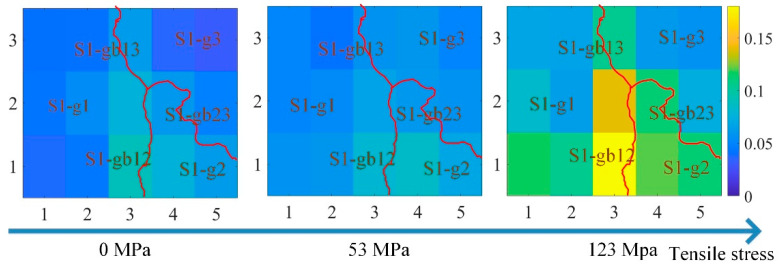
The images for the RMS of the time-response histograms at different locations to reflect the inhomogeneous magnetic properties at different locations. The size of each image is 16.2 mm × 13.6 mm.

**Figure 12:**
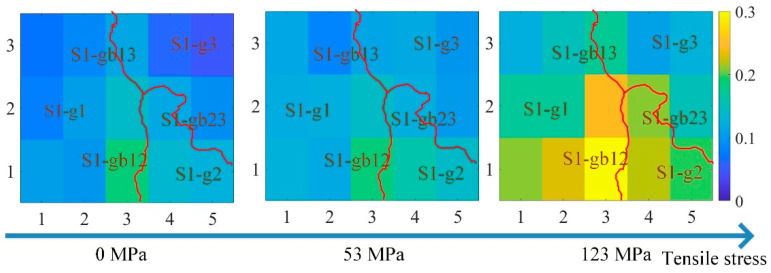
The images for the peak value of the time-response histograms at different locations to reflect the inhomogeneous magnetic properties at different locations. The size of each image is 16.2 mm × 13.6 mm.

However, due to the grain and grain boundary, the material properties at different locations were not uniform. Figure 9 shows the curve of the three features of the time-response histograms on S1-g1, S1-g3, and S1-gb13. Figure 10, Figure 11 and Figure 12 show the two-dimensional images for RSM, mean, and peak value of the time-response histograms at different locations. The mean, RMS, and peak values had the same increasing tendency with the increase of stress. However, these features were higher on the grain boundaries than on the grains. A clear positive correlation between the measured tensile stress state and the corresponding MBN was verified. The 180° DW motion inside the grain and 90° DW motion around the grain boundary [46] were directly proportional to the stress fluctuation, while the time-response histogram features reflected the variation of DW motion with stress. As shown in Figure 9, Figure 10, Figure 11 and Figure 12, the grain boundaries were more susceptible to external tensile stress, especially since the time-response histogram was much higher on the grain boundary S1-gb12. A minor failure was more likely to occur on the grain boundary S1-gb12 [9,10].

MBN emission increased along with tensile stresses and decreased with compressive stresses, especially for uniaxial loading and elastic regimes [13]. The total tensile stress acting normal to the grain boundary during elastic deformation was a sum of the internal stress due to the applied stress, resolved onto the grain boundary plane [9]. The domain-wall motion in this case was a function of microstresses (the function of grain and grain-boundary microstructure). The microstress was higher on the grain boundary than on the grain, making the grain boundary more prone to early minor failure formation [8]. The time-response histograms and their features characterized time characteristics of DW motion to quantitatively analyze the inhomogeneous material’s properties affected by the stress, grain, and grain boundary.

### 4.2. The Difference Elimination of Magnetic Properties before the Tensile Test

**Figure 13:**
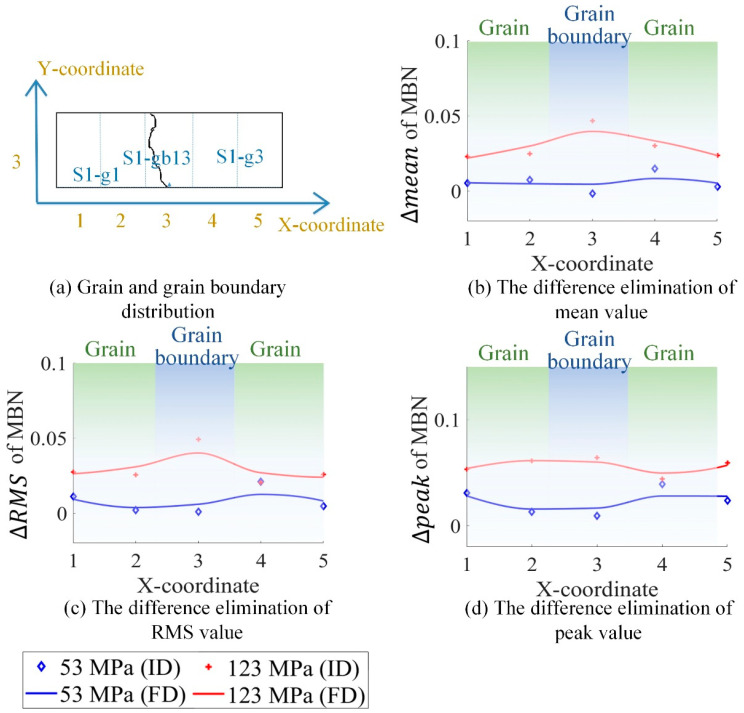
The different elimination of magnetic properties before the tensile test. FD denotes the fitting data, and ID denotes the original data.

Due to the residual stress and material manufacturing, the magnetic properties under 0 N exhibited a small difference, as shown in Figure 9, Figure 10, Figure 11 and Figure 12. It was better to reduce the difference before the tensile test to analyze the variation of the inhomogeneous material’s properties affected by the stress, grain, and grain boundary, as shown in Figure 13.

The difference between these features with stress and without stress is defined in the following, which was extracted from Equations (10)–(12):

(13) ΔRMS=RMS(σ)−RMS(0)

(14) Δmean=mean(σ)−mean(0)

(15) Δpeak=peak(σ)−peak(0)

where RMS(σ),  mean(σ), and peak(σ) denote RMS, mean, and peak value with stress, respectively; and RMS(0),  mean(0), and peak(0) denote RMS, mean, and peak value without stress, respectively. From Figure 13, ΔRMS, Δmean, and Δpeak were almost uniform at the different locations when the tensile stresses increased from 0 MPa to 53 MPa. This means that ΔRMS, Δmean, and Δpeak removed the difference in magnetic properties before the tensile test. When the tensile stresses increased from 0 MPa to 123 MPa, ΔRMS, Δmean, and Δpeak on the grain boundaries were higher than on the grains. The time-response histogram on the grain boundary was more susceptible to the tensile stress; when the tensile stress increased 123 MPa, stress made the degree of the material properties’ inhomogeneity much more significant on the grain boundary than on the grain. ΔRMS, Δmean, and Δpeak reduced the influence of the material microstructure on the initial material state before the tensile test, reflecting the inhomogeneity of the material properties on the grain and grain boundary affected by stress.

### 4.3. Time-Division to Separate the Magnetic Properties’ Variation Affected by Stress, Grain, and Grain Boundary

Due to stress transfer on the grain and grain boundary, the magnetic properties and material properties were inhomogeneous [11]. The time-division was carried out to optimize the time interval to separate the material properties’ variation affected by the grain and the grain boundary.

**Figure 14:**
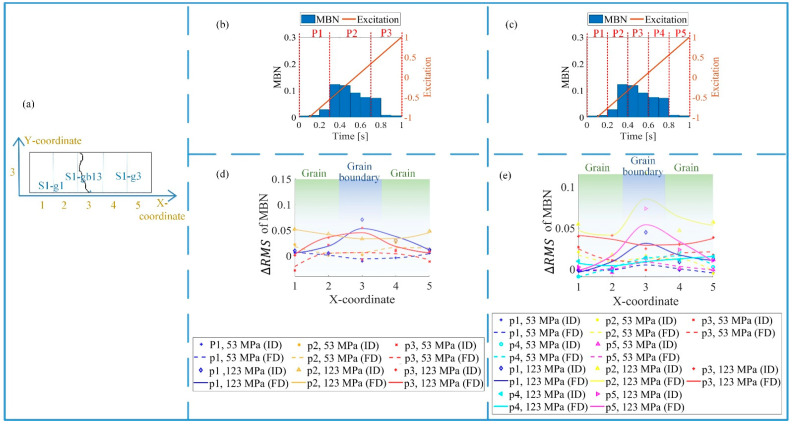
Two methods for time-division. (**a**) Grain distribution; (**b**) time-division with three time bands; (**c**) time-division with five time bands; (**d**) MBN features with three time bands; (**e**) MBN features with five time bands.

The main factor of time-division is to the method of localizing the time bands [21]. The time-division had a high resolution and less redundant information for the material properties’ variation separation. From Figure 14, there are two methods for time-division for the RMS feature, which are the time-division with five time bands and the time-division with three time bands. In this study, the optimal time-division was in line with the excitation frequency. From Figure 4 and Figure 14, it was found that the bins from 0.1 s to 0.3 s and from 0.7 s to 0.9 s highlighted the different magnetic properties inside the grain and around the grain boundary. Based on the time-response histograms at different locations shown in Figure 14, the time-division with three time bands was enough to separate the material properties affected by stress, grain, and grain boundary. The time bands p1 (0–0.3 s) and p3 (0.7–1 s) highlighted the 90° DWs around the grain boundary, which identified the material properties’ variation affected by the tensile stress and microstructure. The time bands p2 (0.3–0.7 s) found 180° DW motion, which determined the material properties’ variation affected by the tensile stress.

**Figure 15:**
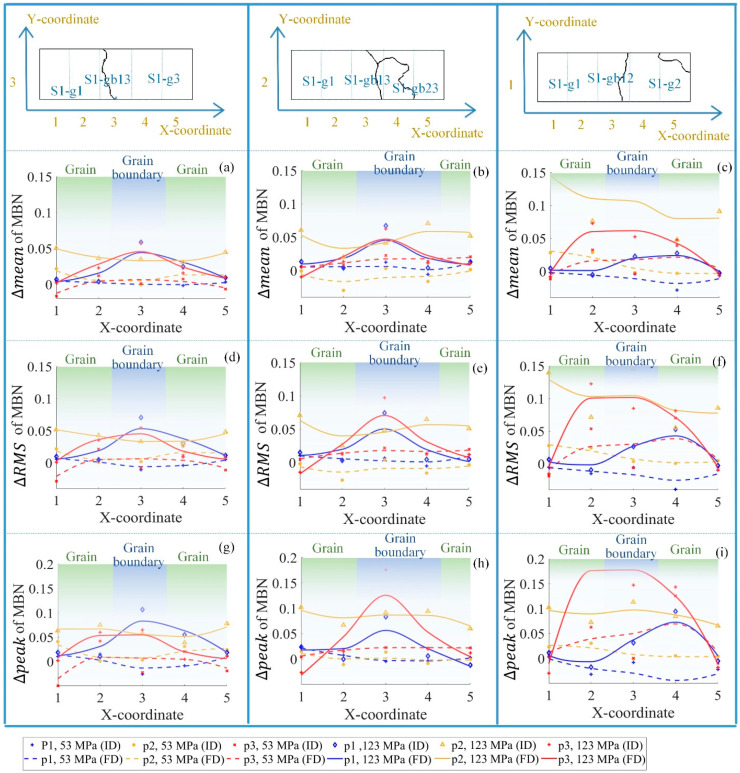
The time-division to separate the magnetic properties affected by stress, grain, and grain boundary. (**a**,**d**,**g**) illustrate the ΔRMS, Δmean, and Δpeak on the S1-g1, s1-g3, and s1-gb13; (**b**,**e**,**h**) illustrate the ΔRMS, Δmean, and Δpeak on the S1-g1, s1-gb13, and s1-gb23; (**c**,**f**,**i**) illustrate the ΔRMS, Δmean, and Δpeak on the S1-g1, s1-g2, and s1-gb12. The histogram features in p1 (0–0.3 s) and p3 (0.7–1 s) identified the variation affected by the tensile stress and microstructure, and the histogram features in p2 (0.3–0.7 s) identified the variation affected by the tensile stress. FD denotes the fitting data, and ID denotes the original data.

As shown in Figure 15, the time-division was used for the feature separation on each part of the sample. For the time bands p1 and p3, the difference between the time-response histogram features *(*ΔRMS, Δmean, and Δpeak) around the grain boundary increased much more than inside the grain under 123 MPa. However, the variation of these features for time band p2 was almost uniform.

When the tensile stress was 53 MPa, the material properties on the grains and the grain boundaries were uniform. When the tensile stress increased to 123 MPa, due to the influence of stress and the material microstructure, material properties on grain boundaries varied significantly, reflected by the time bands p1 and p3. However, for time band p2, the variation in the time-response histogram features was almost uniform from 53 MPa to 123 MPa.

The optimal time-division was based on the correlation between the applied field and the DW motion on different locations. When the stress increased, the $90^{{}^{\circ}}$ DW appeared around the grain boundary. It took more time on the grain boundary to reach magnetic saturation during the magnetization process. As a result, the MBN activities lasted longer, and the time-response histogram features were larger on the grain boundary. After the difference elimination of material properties before the tensile test, the time-division separated the material properties affected by the grain and the grain boundary. The time bands p1 and p3 highlighted the different variations of micro- and macromagnetic properties on the grain and grain boundary under stress, while the time band p2 removed the variation of material properties affected by the microstructure under stress.

In order to further analyze the sensitivity of the time-division for the material properties’ variation separation with different stress states directly, contrast values [49] were extracted from RMS, mean, and peak at the time intervals P1, P2, and P3. Contrast value is defined as:

(16) $$contrast=\sqrt{\frac{{\left(\sum_{x=0}^{N-1}I_{m}\left(x\right)-\bar{I_{m}}\right)}^{2}}{N}}$$

where $I_{m}\left(x\right)$ denotes the curve of time-response histogram features $\bar{I_{m}}$ denotes the mean of $I_{m}\left(x\right)$, N denotes the max of X-coordinate, and m and n denote the value of Y-coordinate and X-coordinate, respectively.

From Table 2, the contrast values of ΔRMS, Δmean, and Δpeak in the time intervals P1 and P3 increased with increment of stress, while these values in the time interval P2 exhibited a minor change. These results for contrast values showed that the time-division separated the variation of magnetic properties affected by the stress, grain, and grain boundary.

The time-response histograms characterized the different time characteristics of the MBN signal inside the grain and grain boundary. The optimal time-division highlighted variations of micro- and macromagnetic properties affected by the grain, grain boundary, and stress. Based on the approach proposed in this paper, the standard sample of the ferromagnetic material without external stress or the residual magnetic field of the sample will be used as a reference to reduce the difference of material properties before tensile test to improve the results of the evaluation of inhomogeneous material properties affected by the grain, grain boundary, and stress [50]. After removal of background information, the different time responses at different locations can analyze inhomogeneity of material properties for ferromagnetic material on a microscopic scale.

### 4.4. Conclusions and Future Work

In this paper, based on the observation of grain and grain-boundary distribution, the time-response histogram of MBN was established to nondestructively characterize silicon steel sheet’s inhomogeneous magnetic properties affected by the grain and grain boundary during elastic deformation. The main conclusions are summarized as follows:

(1) The time-response histogram characterized the difference in DW motion at different locations. The time-response histogram became different on the grain and grain boundary with increased stress, characterizing the variation of the inhomogeneous magnetic properties under stress.
(2) The varying degrees of magnetic properties are much higher around the grain boundary than the grain interior with the stress state. In particular, MBN signals around the grain boundary S1-gb12 were much higher than at other locations, reflecting the fact that the grain boundaries were more susceptible to external tensile stress.
(3) The RMS, mean, and peak were extracted from the time-response histograms to quantify the magnetic properties’ variation on the grain and grain boundary. These three features had the same tendency at different locations under stress.
(4) After difference elimination of magnetic properties before the tensile test, the time-division was carried out to extract the RMS, mean, and peak in the optimized time interval, which separated the variations of magnetic properties affected by stress, grain, and grain boundary. The optimal time intervals highlighted the different variations in magnetic properties affected by the microstructure and stress, respectively.

This study provides a theoretical and experimental basis for a better understanding of the effect of grain and grain boundary on the evaluation of inhomogeneous material properties for other ferromagnetic material (especial Q235, steel. etc.) by using MBN [51,52]. The time-response histogram of the MBN signal can analyze the variation of magnetic properties on the grain and grain boundary during plastic deformation, especially the variation in inhomogeneity of magnetic properties before crack formation on a microscopic scale [2]. The high-sample-rate acquisition method improves the time to resolve DW motion detection, and the high-spatial-resolution magnetic probe improves the resolution for the characterization of the inhomogeneous material’s properties on the microscopic scale.

## Figures and Tables

**Table 1:** Chemical composition of silicon steel sheets expressed as weight percentages.

Fe	Si	C	Mn	P	S	Al
Balance	3.00~5.00	0.06	0.15	0.03	0.25	5.10~8.50

**Table 2:** The contrast value of ΔRMS, Δmean, and Δpeak in the time intervals P1, P2, and P3.

		53 MPa			123 MPa		
Location	Feature	P1	P2	P3	P1	P2	P3
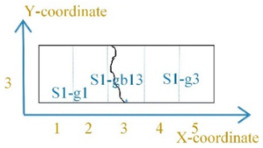	Δmean	0.0059	0.0079	0.0081	0.0202	0.0071	0.0191
	ΔRMS	0.0068	0.0102	0.0125	0.0241	0.0085	0.0201
	Δpeak	0.0155	0.0157	0.0169	0.0361	0.0141	0.0279
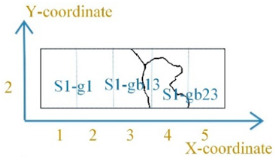	Δmean	0.0071	0.0101	0.0066	0.0236	0.0174	0.0238
	ΔRMS	0.0061	0.0097	0.0061	0.0272	0.0158	0.0381
	Δpeak	0.0112	0.0109	0.0071	0.0355	0.0164	0.0702
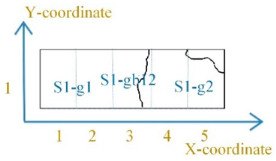	Δmean	0.0096	0.0121	0.0167	0.0293	0.0133	0.0329
	ΔRMS	0.0124	0.0114	0.0177	0.0364	0.0184	0551
	Δpeak	0.0179	0.0121	0.0248	0.0392	0.0181	0.0981

## Data Availability

The data presented in this study are available on request from the corresponding author. The data are not publicly available as the data forms part of an ongoing study.
